# Roll-to-roll slot-die coating of 400 mm wide, flexible, transparent Ag nanowire films for flexible touch screen panels

**DOI:** 10.1038/srep34322

**Published:** 2016-09-28

**Authors:** Dong-Ju Kim, Hae-In Shin, Eun-Hye Ko, Ki-Hyun Kim, Tae-Woong Kim, Han-Ki Kim

**Affiliations:** 1 Kyung Hee University, Department of Advanced Materials Engineering for Information and Electronics, 1 Seocheon, Yongin, Gyeonggi-do 446-701, Republic of Korea; 2 Dynamic Korea Technology, R&D Center, 116-60, Sanho-daero, Gumi City, Gyeong-Buk, 39377, Republic of Korea; 3 Samsung Display, OLED R&D Center, Yongin, Gyeonggi-do 446-711, Republic of Korea

## Abstract

We report fabrication of large area Ag nanowire (NW) film coated using a continuous roll-to-roll (RTR) slot die coater as a viable alternative to conventional ITO electrodes for cost-effective and large-area flexible touch screen panels (TSPs). By controlling the flow rate of shear-thinning Ag NW ink in the slot die, we fabricated Ag NW percolating network films with different sheet resistances (30–70 Ohm/square), optical transmittance values (89–90%), and haze (0.5–1%) percentages. Outer/inner bending, twisting, and rolling tests as well as dynamic fatigue tests demonstrated that the mechanical flexibility of the slot-die coated Ag NW films was superior to that of conventional ITO films. Using diamond-shape patterned Ag NW layer electrodes (50 Ohm/square, 90% optical transmittance), we fabricated 12-inch flexible film-film type and rigid glass-film-film type TSPs. Successful operation of flexible TSPs with Ag NW electrodes indicates that slot-die-coated large-area Ag NW films are promising low cost, high performance, and flexible transparent electrodes for cost-effective large-area flexible TSPs and can be substituted for ITO films, which have high sheet resistance and are brittle.

Rapid advances in flexible mobile phones, tablets, labtops, informative flat panel displays, and navigation systems for curved dash-boards in automobiles have increased demand for thin, light, transparent, highly responsive, and low-cost flexible touch screen panels (TSPs)^1^,^2^,^3^. Because flexible TSPs are one of the key components of high-performance flexibility displays, great effort has been focused on the development of TSPs consisting of flexible materials. To meet the advanced standards for flexible TSPs, it is imperative to develop high-quality and cost-effective transparent electrodes because the responsivity, clear visibility under various ambient light conditions, mechanical flexibility, and cost of flexible TSPs are critically dependent on the electrical, optical, and mechanical properties as well as fabrication process of transparent conductive electrodes (TCEs). The most widely used TCEs in resistive- or capacitive-type TSPs are Sn-doped In_2_O_3_ (ITO) films prepared by a vacuum-based sputtering process^4^,^5^. Although sputtered ITO films have a high conductivity and transparency in the visible region, critical problems such as the high sheet resistance of thin ITO films, the scarcity of indium resources, and the brittleness of ITO film make it impractical to use ITO films for large-area and cost-effective flexible TSPs^6^. In particular, the high sheet resistance (100–150 Ohm/square) of thin ITO film (20 nm) is a critical limit for realization of large-area capacitive-type TSPs above 20 inches. The need to replace high-cost and high-resistance ITO films with better performing TCE materials has yielded several TCE materials such as carbon nanotube (CNT) networks^7^,^8^, graphene sheets^9^,^10^,^11^, conducting polymer films^12^,^13^, metal nanowires^14^,^15^,^16^,^17^,^18^,^19^, as well as metal grids^20^,^21^. However, TSPs containing CNTs, graphene, and PEDOT:PSS electrodes displayed only modest performance due to the relatively high sheet resistance of CNTs and graphene and the instability of acidic PEDOT:PSS electrodes. Recently, metal grid TCEs were employed in large-area TSPs due to their low sheet resistance and high transmittance^22^. However, the Moiré effect, which is caused by a set of grid lines, in addition to photo-lithography-based complications and a high-cost patterning process are limitation of metal grid TCEs. Metal (Ag or Cu) nanowire (NW) percolating network films are being intensively investigated in academia and industry as promising TCEs for large-area flexible TSPs because of their simple and cost-effective printing process, metallic low resistivity, good flexibility, and absence of the Moiré effect^23^,^24^,^25^,^26^. Li *et al.*, reported capacitive touch pads fabricated on paper using high-concentration Ag nanowire ink^27^. Lee *et al.,* also demonstrated flexible TSPs on Mayer-rod coated PEDOT:PSS/Ag NW hybrid electrodes^28^. They demonstrated the feasibility of cost-effective Ag NW electrode for TSPs on non-flat surfaces^27^,^28^,^29^. Although the performance characteristics of Ag NW films prepared by brush painting, Mayer rod coating, spray coating, spin-coating, and a transfer process have been well studied, continuous RTR slot-die coating of Ag NW ink and application of Ag NW film on large-area flexible TSPs have not been investigated. In particular, it is important to develop a method for continuous two-step slot-die coating of Ag NWs. Furthermore, Ag NWs synthesized for use in commercialized ITO electrodes should be covered by a coating layer to protect them from sulphur in the atmosphere.

In this work, we report the electrical, optical, and mechanical properties of 400 mm wide Ag NW percolating network electrodes prepared using a continuous RTR slot-die coater under atmospheric conditions. Based on our understanding of Ag NW ink flow at the lip of the slot die, we designed a slot die head appropriate for Ag NW ink and controlled the density of Ag NWs on the PET substrate. By continuous two-step RTR slot-die coating, we fabricated highly transparent and conductive Ag NW network films covered uniformly by over-coating layer to protect the Ag NWs. Capacitive-type flexible TSPs with diamond-patterned Ag NW electrodes were successfully operated, thereby demonstrating the feasibility of using the cost-effective Ag NW percolating network electrodes described here as alternative to conventional high-cost ITO electrodes.

## Results

**Figure 1:**
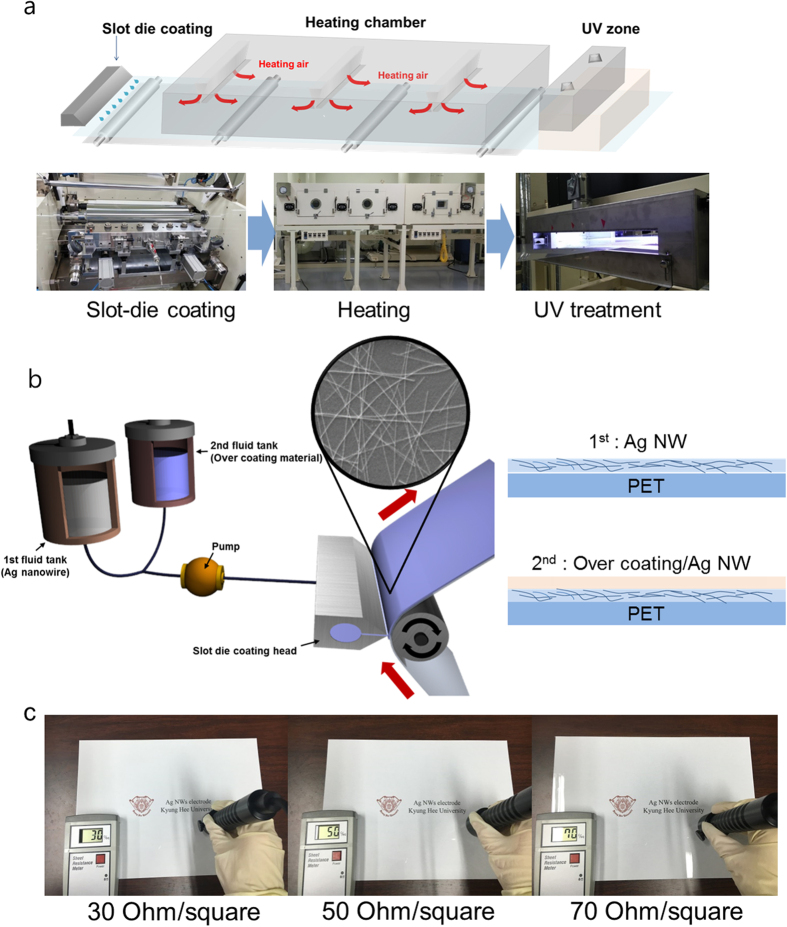
(**a**) Schematic illustration of the continuous RTR slot-die coating system used to coat Ag NWs and over coating layer on the PET substrate. The RTR slot-die system consisted of a slot-die coating zone, heating zone, and UV treatment zone. (**b**) Schematics of slot-die coating of Ag NWs and the over-coating layer. (**c**) Picture of Ag NW percolating network electrodes with different sheet resistances (30 to 70 Ohm/square).

Schematics of a pilot-scale RTR slot-die coating system (DKT 2015-R1-SHU500) used to coat Ag NWs and an over-coating layer onto 125 μm-thick PET substrate (TORAY ADVANCED MATERIALS KOREA INC., supporting Table S1) are provided in Fig. 1a. The RTR slot die coating system consisted of ink tanks, a slot die coating zone, a substrate heating zone, and a UV treatment zone. Using the RTR slot die coating system (Supporting information Figures S1 and S2), the Ag NW layer was uniformly coated on the PET substrate through the slot die head, and then the films passed through the heating zone (120 °C) by means of unwinding and rewinding the roller at a constant speed of 2 m/min. Figure 1b exhibits slot die coating of Ag NWs on the moving PET substrate at room temperature. After coating of the Ag NWs, the over coating layer was also uniformly coated on the Ag NW layers. Ag NWs covered by the over-coating layer then entered the heating zone (80 °C) and UV treatment zone filled with nitrogen ambient by means of unwinding and rewinding the roller at a constant speed of 2 m/min. The purpose of the over-coating layer was to impart mechanical strength to the films and protect the Ag NWs layer from direct environmental exposure. As discussed by Yacaman, atmospheric corrosion of Ag NWs resulted in the formation of silver sulphide nanocrystals on the surfaces of Ag NWs^30^. Therefore, an effective over-coat layer is to protect against atmospheric corrosion to enable commercialization of slot-die-coated Ag NW network electrodes. For simplicity, we refer to the Ag NWs covered by the over-coating layer as OC-Ag NW films hereafter. Figure 1c shows the *in-situ* measured sheet resistance of OC-Ag NW films fabricated using a RTR slot-die coater as a function of pump frequency used to feed the Ag NW ink to control the density of the Ag NW network.

**Figure 2:**
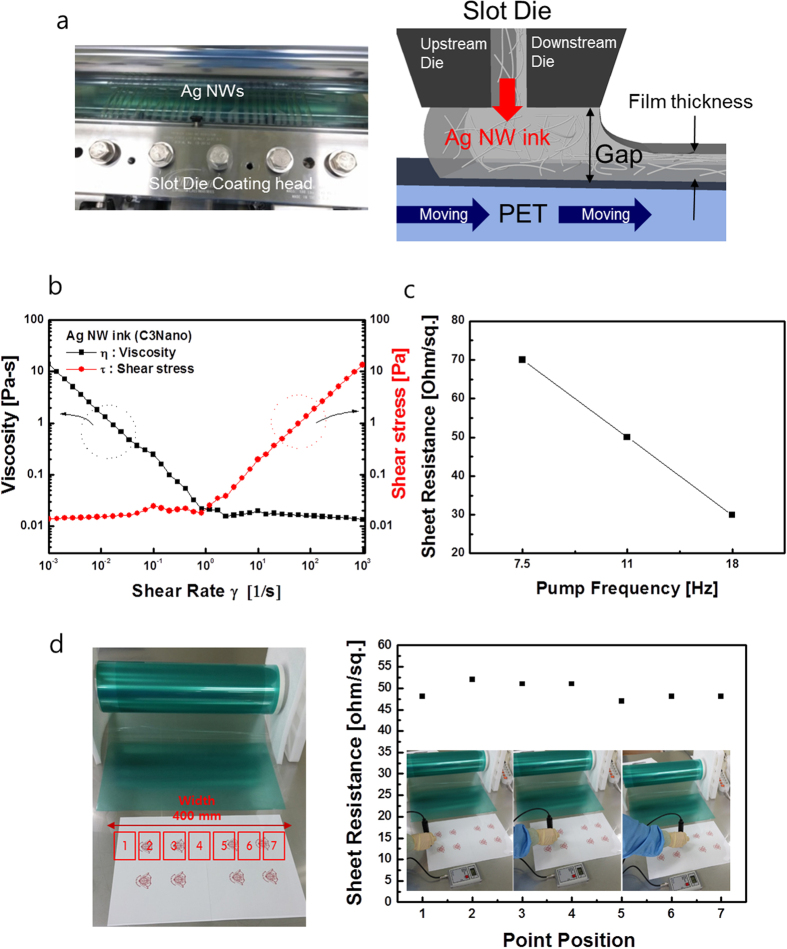
(**a**) Picture of the slot die head ejecting Ag NW inks on the PET substrate and schematic structure of a slot die head used for Ag NW coating. (**b**) Viscosity and shear stress of Ag NWs ink versus shear rate. (**c**) Sheet resistance of the slot-die coated Ag NW network electrode as a function of increasing pump frequency for feeding Ag NW ink. (**d**) Picture of OC-Ag NW films and sheet resistance of Ag NWs at different positions indicating good uniformity of the Ag NW network films.

To fabricate large area TSPs and confirm the operation uniformity of flexible TSPs, uniform coating of Ag NW ink is very important. Therefore, proper design of the slot die to unsure uniform distribution of Ag NW ink is critical for the fabrication of large area Ag NW network films with uniform sheet resistance. Figure 2a shows a picture and the schematic structure of the slot die head in the RTR slot-die coating system. In the slot die coating process, the Ag NW ink is pumped from the Ag NW ink tank to a die and distributed across the width of a narrow slot. The Ag NW ink filled the gap between the die lips and moving PET substrate and formed Ag NW network films. The thickness of the Ag NW film was controlled by the Ag NW ink flow rate to the slot die and the speed of the moving PET substrate (Figure S2). Right schematic in Fig. 2a illustrates the flow of Ag NW ink at the exit of the slot die. To optimize the design of the slot-die head for Ag NW ink, we measured the viscosity of Ag NW ink as a function of shear rate. The viscosity and shear stress of Ag NW ink as a function of shear rate were analyzed using Rheoplus Software as shown in Fig. 2b. The viscosity of Ag NW ink decreased with increasing shear rate (shear thinning)^31^,^32^. Therefore, the ink acted as a non-Newtonian fluid. The uniformity of the slot-die coated Ag NW ink was critically affected by the PET substrate moving speed, the viscosity of the Ag NW ink, and the geometry of the upstream and downstream lips. Based on use of Ag NW ink with non-Newtonian behaviour, we designed a slot die head for our pilot-scale RTR slot die coater. Under optimized slot-die coating and substrate heating conditions, we achieved uniformly coated Ag NW network films with different sheet resistances as shown in Fig. 2c. In general, the sheet resistance of Ag NWs network is closely related to the density of Ag NWs. Therefore, an increase in the pumping frequency of Ag NW ink into the slot die head led to a decrease in sheet resistance from 70 to 30 Ohm/square, as shown in Table 1. Figure 2d shows a picture of 400 mm wide OC-Ag NW network films obtained after continuous two-step slot die coating. Sheet resistance was measured *in-situ* in the direction of width. Due to the uniform distribution of Ag NWs in the lips and the constant PET velocity and tension, the RTR slot-die coated Ag NW network films on the 400 mm-wide PET substrate showed good uniformity regardless of position. The inset picture in Fig. 2d shows the *in-situ* sheet resistance of a RTR slot-die-coated Ag NW network electrode with a sheet resistance of 50 Ohm/square.

**Figure 3:**
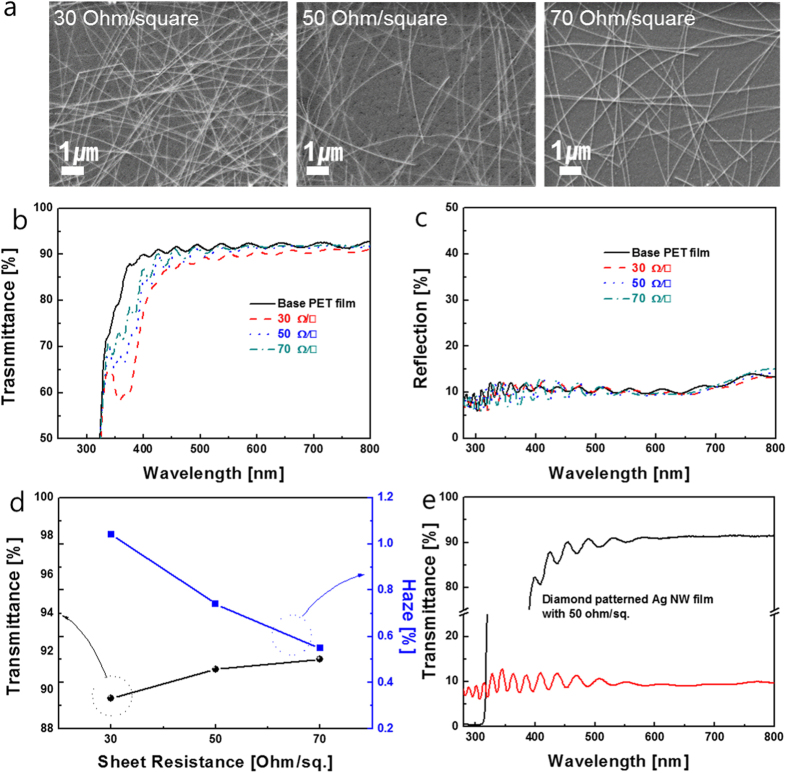
(**a**) Surface FESEM images of RTR slot-die coated Ag NW networks as a function of pumping frequency. (**b**) Optical transmittance and (**c**) reflectance of RTR slot-die-coated Ag NW network films with decreasing Ag NW densities. (**d**) Haze and transmittance at 550 nm wavelength region of RTR slot-die-coated Ag NW network films. (**e**) Optical transmittance and reflectance of diamond-patterned Ag NW film.

Surface FESEM images of slot-die coated Ag NW network films as a function of pumping frequency are shown in Fig. 3a. It is clear from these images that the coverage of Ag NWs on the PET substrate was mainly affected by the pumping frequency used to feed the Ag NW ink into the slot die. As expected from the sheet resistance of Ag NW films in Fig. 2c, an increase in pumping frequency led to an increase in percolating Ag NW density and a decrease in sheet resistance of the Ag NW network films. Figures 3b show the optical transmittance the OC-Ag NW network films with different sheet resistances ranging from 30 to 70 Ohm/square. All RTR slot-died coated Ag NW network films had a high optical transmittance and low reflectance in the visible wavelength region, as well as a broad surface plasmon resonance band in the UV region^33^. Compared to OC-Ag NW network films with sheet resistances of 50 and 70 Ohm/square, respectively, the Ag NW network film with a sheet resistance of 30 Ohm/square showed slightly lower optical transmittance due to scattering by the high-density Ag NWs. OC-Ag NW films with a sheet resistance of 70 Ohm/square had the highest transmittance of 90% and the lowest reflection of 10% at a wavelength of 550 nm. Although the increase in Ag NW density led to a decrease in optical transmittance, all RTR slot-die coated Ag NW network films exhibited optical transmittance in the visible wavelength region that was high enough for use in flexible TSPs. In addition, the optical transmittance of the Ag NW networks was higher than that of semi-transparent Ag film, because the high transmittance of Ag NWs is achieved by passing of light through the Ag NW uncovered region. As shown in Fig. 3c, the low reflectance of the Ag NW network regardless of the sheet resistance indicates that light reflection by Ag NWs is negligible due to their very narrow width (~22 nm). Figure 3d shows haze and optical transmittance values of the Ag NW network films with different sheet resistances. Haze of TCEs is defined as the percentage of transmitted light passing through the film that deviates more than 2.5° from the incident beam by forward scattering^1^:


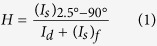


Here, I_d_ is the light flux transmitted directly, and (I_s_)_f_ is the flux that undergoes forward scattering, i.e. scattering intensity between 0° and 90°. As discussed by Mini *et al.,* TCEs should have a low haze value for successful integration of flexible TSPs into flexible displays, because the blurriness of TSPs is mainly related to the haze value of the TCE film^17^. The recommended haze value of TCEs used in high performance TSPs is less than 1%. The diamond-shape patterned Ag NW electrodes showed high optical transmittance and low reflectance in addition to acceptable haze values. As shown in Table 1 and Fig. 3d, the haze of the OC-Ag NWs layer is lower than that of pure Ag network due to the existence of the over coating layer, which uniformly covered the slot-die coated Ag NWs network and improved the flatness of the OC-Ag NWs network. Therefore, reduced light scattering on the OC-Ag NWs decreased the haze value of the OC-Ag NWs network films^17^,^22^. In addition, due to the high optical transmittance of patterned OC-Ag NW network films as shown in Fig. 3e, flexible TSPs consisting of merged Ag NW network films showed a high an optical transmittance of 90% and a low reflectance of 10%. Table 1 summarized the electrical and optical properties of slot-die coated Ag NW network film as a function of pump frequency.

**Figure 4:**
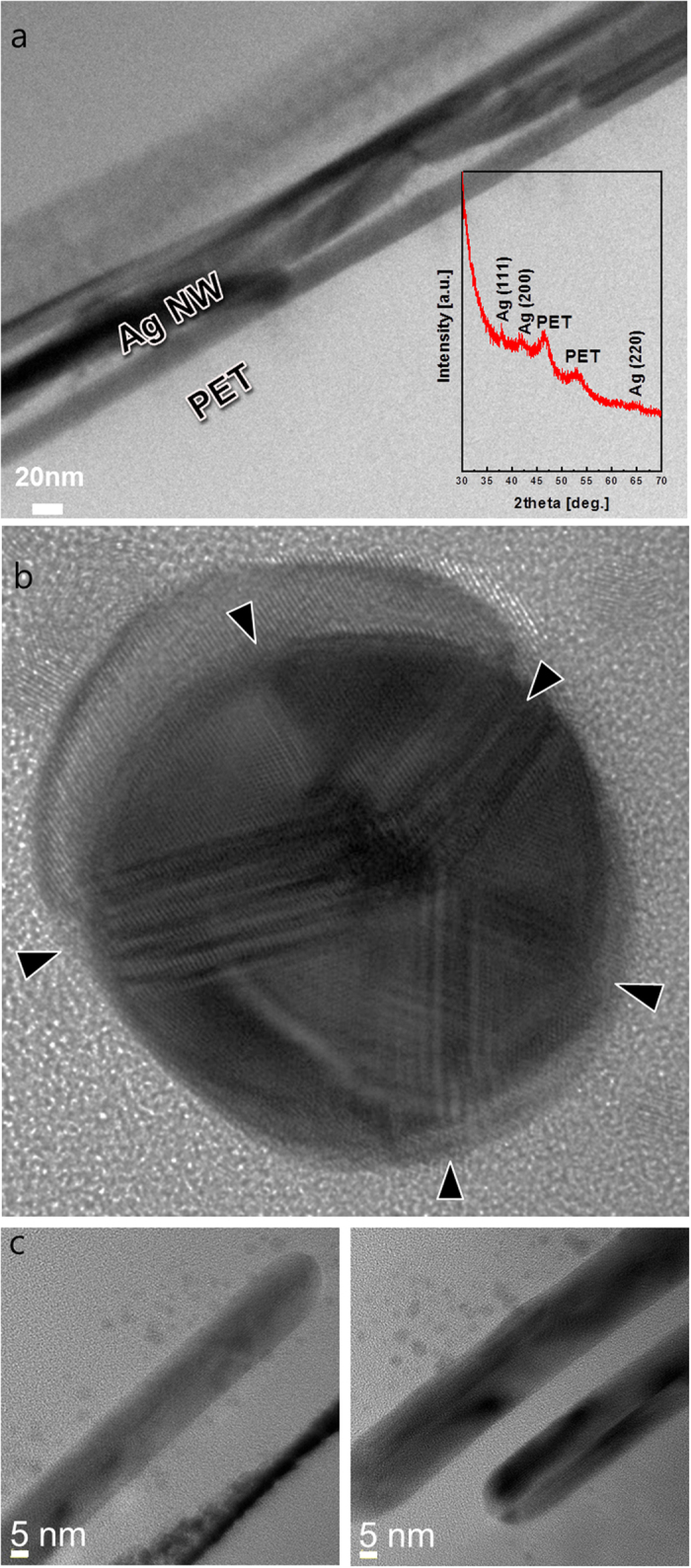
(**a**) Cross-sectional TEM image of RTR a slot-die coated Ag NW network on PET substrate. (**b**) High resolution TEM image of the end of a single Ag nanowire showing five distinct boundaries. (**c**) Enlarged TEM image of the side plane of Ag nanowires with diameters of around 18.7 and 22.6 nm, respectively.

**Figure 5:**
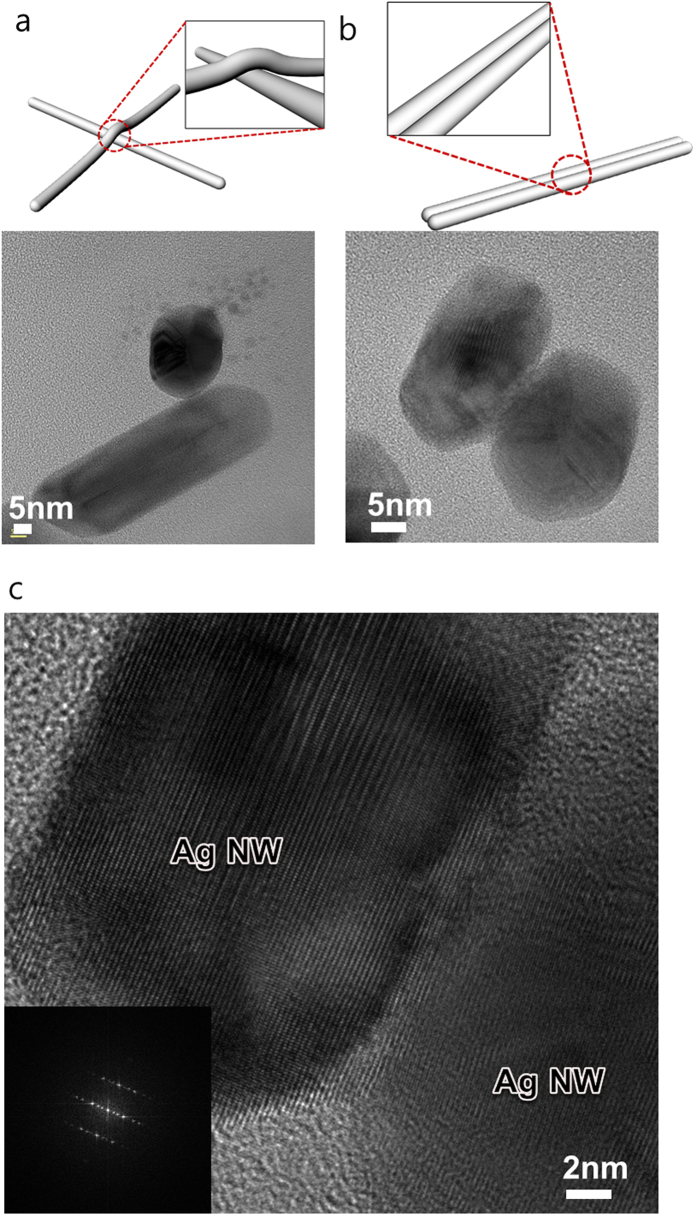
Cross-sectional TEM image of (**a**) a cross-bar junction and (**b**) laterally parallel junction between Ag NWs. (**c**) High resolution TEM image of the (100)-(100) side plane junction between Ag NWs.

Cross-sectional TEM images of RTR slot-die-coated Ag NW network film with a sheet resistance of 50 Ohm/square on PET substrate in addition to an XRD plot insert are shown in Fig. 4a. The slot-die coated Ag NWs were randomly distributed on the PET substrate with smooth surface morphology. As shown in the XRD plot in inset of Fig. 4a, the Ag NWs were terminated by the (111) plane and the (100) side surface plane. Both the (111) end plane and (100) side surface plane of the Ag NWs were physically connected and provided conduction paths for electrons. In particular, the slot-die coated Ag NWs were parallel to the PET substrate. The enlarged TEM image of a cross-section of an Ag NW in Fig. 4b shows the circle-shaped end of an Ag NW and a pentagonal rod with five boundaries, as indicated by arrows. As discussed by Chen *et al.*, anomalous contrast fringes in boundaries indicate the presence of stacking faults and twinning boundaries^34^. Cross-sectional images of single Ag NW with diameters of around 18.7 around 22.6 nm, respectively, are shown in Fig. 4c. The enlarged images shows that the single Ag nanorod had a round-shaped end. Figure 5 shows TEM images obtained from Ag NW-Ag NW junction regions. As shown in Fig. 5a,b, the junctions between Ag NWs were cross-bar and laterally parallel junctions. The cross-sectional HRTEM image revealed that the (100) side surface plane of the Ag NW contacted the other (100) side surface plane of the Ag NW. Considering percolating Ag NW networks and the optical transparency of the uncovered Ag NW region, a cross-bar junction is more advantageous for decreasing the sheet resistance of Ag NW film, because the cross-bar junctions of Ag NWs provide effective conduction paths for randomly distributed Ag NWs. The enlarged TEM image in Fig. 5c shows that the (100) surface of the Ag NW side plane was well connected to other surface (100) plane of the Ag NW.

**Figure 6:**
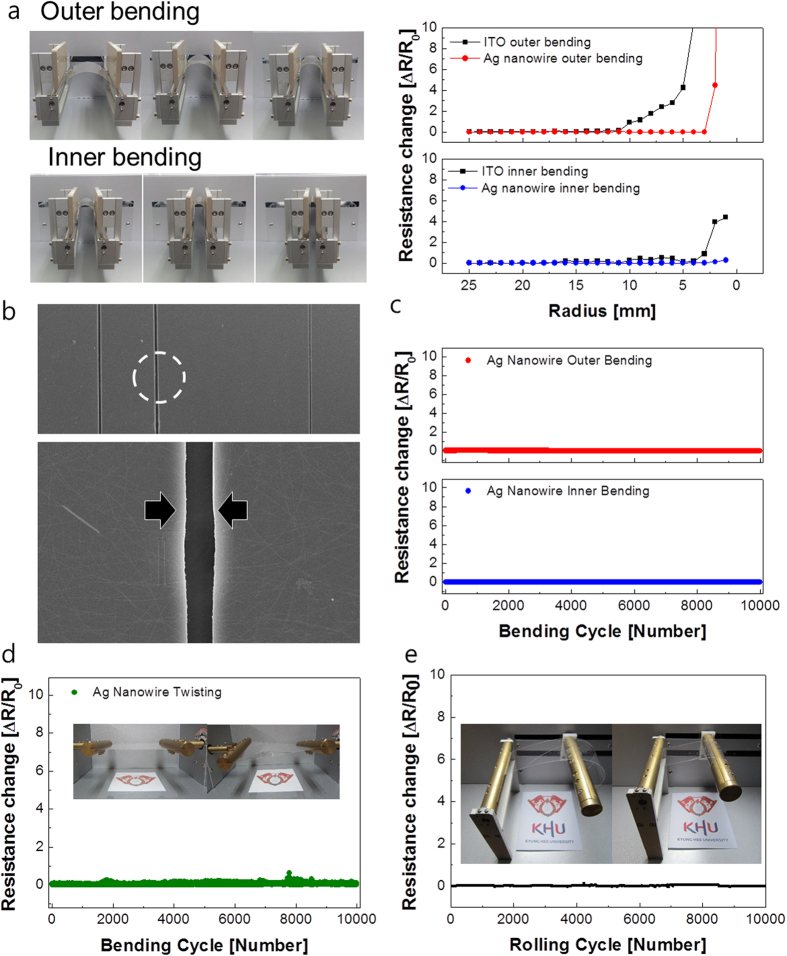
(**a**) Picture of the outer and inner bending test system and resistance changes during outer and inner bending as a function of bending radius. (**b**) Surface FESEM image of OC-Ag NW network film after a 3 mm radius outer bending test. Arrows indicate the regions separated by cracks. (**d**) Dynamic outer and inner bending fatigue tests of OC-Ag NW network films at a fixed bending radius of 5 mm. (**d**) Twisting test of the OC-Ag network film at a twist angle of 10° with inset picture showing twisting steps. (**e**) Rolling test of OC-Ag network films with increasing rolling cycles; the inset picture shows the rolling step.

To demonstrate the feasibility of Ag NW network films as flexible TCEs for flexible TSPs, we investigated their mechanical flexibility based on specially designed bending test systems. The left pictures in Fig. 6a shows the outer/inner bending systems that we used to measure changes in resistance of various outer/inner bending radii. As shown on the right in Fig. 6a, the change in resistance of an Ag NW network film can be expressed as (R-R_0_)/R_0_, where R_0_ is the initial measured resistance and R is the resistance measured under substrate outer/inner bending^35^,^36^. The outer bending test results revealed that the slot-die coated Ag NW network film kept a constant resistance until the bending radius reached 2.5 mm. However, a further decrease in the outer bending radius rapidly increased the resistance changes due to tearing of the OC-Ag NW layer, as shown in Fig. 6b. Compared to conventional ITO film with a critical outer bending radius of 8–10 mm, the slot-die coated Ag NW network film showed superior flexibility due to the high strain failure of Ag. In the inner bending tests, the Ag network film also showed a constant resistance change until the sample was bent to an inner bending radius of 2 mm (the bending limit). Even though the OC-Ag NW network films delaminated from the PET substrate or many cracks formed in the OC-Ag NW network films under severe inner bending, the change in resistance was smaller than that observed during the outer bending test. Under inner bending, the flexible Ag network film showed small resistance changes because of the overlapping of cracked or delaminated layers. However, when outer bending was applied, the Ag network films were under tensile stress, as shown in the inset of Fig. 6a. Due to this tensile stress, cracks isolated the OC-Ag network and increased the resistance change when it was severely bent below the bending radius of 7 mm, as shown in the surface FESEM image of the OC-Ag network after a 3 mm outer bending test. Cracks parallel to the outer bending direction separated the OC-Ag network films. Figure 6c shows the dynamic outer and inner bending test results of OC-Ag network films as a function of increasing bending cycles for a fixed outer and inner bending radius of 5 mm, which is the requested bending radius in flexible TSPs. Both dynamic outer and inner bending fatigue tests showed no change in resistance (ΔR) after 10,000 bending cycles, demonstrating the good mechanical flexibility of the slot-die coated Ag network films. Figure 6d shows resistance changes of the OC-Ag network film during a twisting test at a fixed twisting angle of 10°. The OC-Ag network film showed constant resistance as the number of twisting cycles increased, indicating that the slot-die coated OC-Ag network films had good flexibility. Figure 6e shows the resistance changes of the OC-Ag network film during the rolling test. The OC-Ag network film was clipped on a rolling bar with a radius of 70 mm as shown in the inset picture, and then rolled repeatedly. During a 10,000 cycle rolling test, the OC-Ag network film showed constant resistance changes. Based on the results of the outer/inner, twisting, and rolling tests, we concluded that the RTR slot-die coated OC-Ag network film had sufficient flexibility to enable realization of flexible TSPs

**Figure 7:**
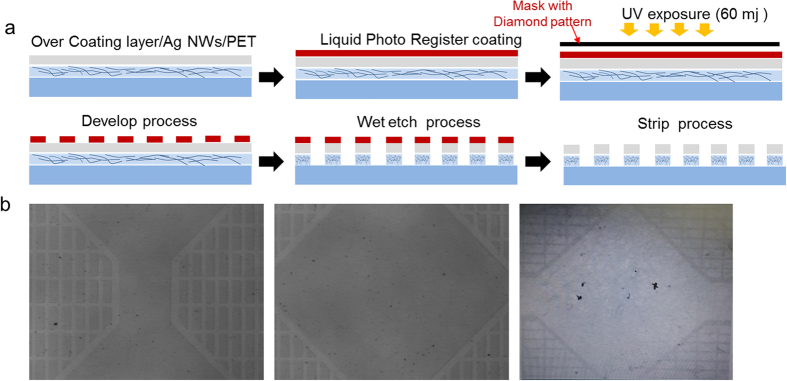
(**a**) Schematic of pattering process of RTR slot-died coated Ag network films. (**b**) Optical microscope images of the diamond-shape patterned Ag network films.

Figure 7a shows the schematic patterning process of RTR slot-die-coated OC-Ag network film based on a wet etching system. By coating a liquid photo resist (LPR) layer and then exposing the positive-masked LPR/OC-Ag NW network/PET films to UV, we successfully patterned OC-Ag NW network films with a typical diamond shape. Optical microscope images of the diamond shape-patterned bottom of Ag NW network films are shown in Fig. 7b. Detailed wet-etching process of the Ag NW film for diamond-patterning was explained in supporting materials Figure S3.

**Figure 8:**
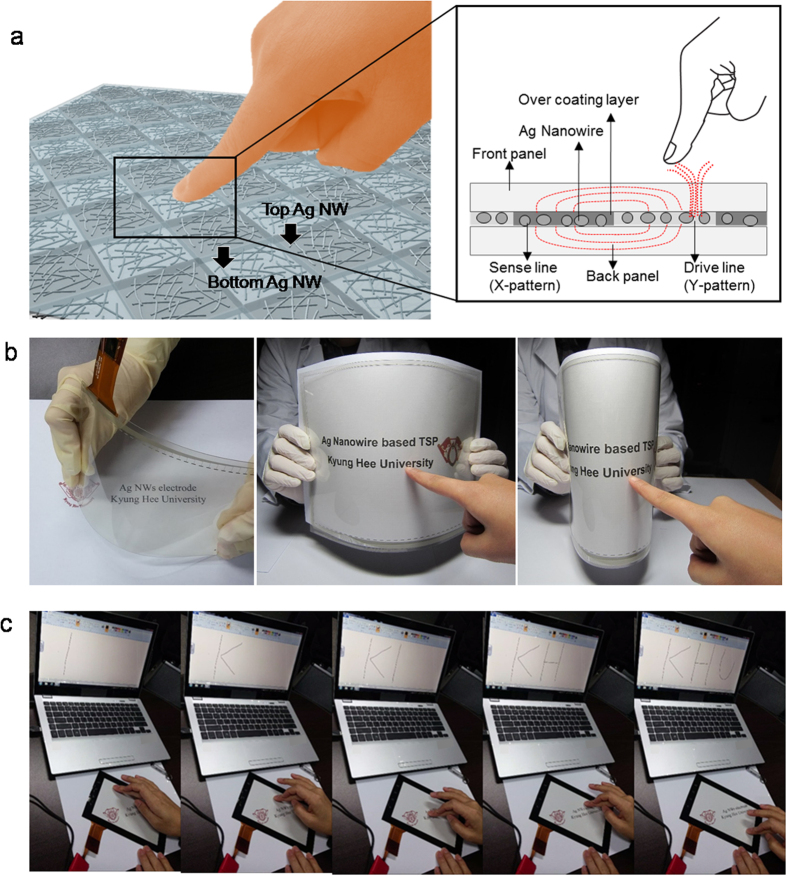
(**a**) Schematics of flexible TSP operation with RTR slot-die coated Ag NW network films. (**b**) Picture of 12-inch curved TSPs with RTR slot-die coated Ag NW network films. (**c**) Operation of TSPs with an Ag NW network electrode.

A schematic structure of flexible TSPs with diamond-shaped top and bottom Ag NW network films is shown in Fig. 8a. By merging the diamond-patterned bottom and top Ag NW network films, we fabricated flexible TSPs with the structure of PET/top Ag NW film/OC-OC/bottom Ag NW film/PET. In general, capacitive-type TSPs are built using two TCEs in parallel, where the patterns of the TCEs form a capacitor holding charge. Touching a finger to the TSPs changes the electric field between the capacitor plates, because human body capacitance absorbs the fringing electric field, as shown in Fig. 8a. Pictures of 12-inch flexible TSPs fabricated on patterned Ag NW electrodes with a low sheet resistance of 50 Ohm/square and high optical transmittance of 90.0% are shown in Fig. 8b. Due to the high optical transmittance of patterned OC-Ag NW network films, flexible TSPs consisting of merged Ag NW network films showed high an optical transmittance as well as good flexibility. By connecting the TSPs to software (MS paint program), we were able to operate the flexible TSP based on the diamond-patterned Ag NW network films. Figure 8c shows the writing function of the flexible TSPs based on diamond-patterned Ag NW network films. Generally, GFF-type TSPs operates by exact sensing of X-Y coordinates and the characteristics of linearity. TSPs with a diamond-shaped Ag NW network films were also operated with protective cover glass. TSPs with diamond-patterned Ag NW network films were successfully used to perform writing functions. This demonstrated that the diamond-patterned Ag NW network films, which had low sheet resistance and high optical transmittance as well as good mechanical flexibility, are a promising transparent, flexible, and cost-effective electrodes that can substitute for conventional ITO electrodes in large area flexible TSPs.

## Conclusion

In summary, we developed a simple RTR slot die coated Ag NW network films for large area flexible TSPs and demonstrated the feasibility of Ag NW electrodes as promising replacements for conventional ITO electrodes. Based on an understanding of Ag NW ink flow and proper slot-die design, we fabricated 400 mm-wide Ag NW network films with a sheet resistance uniformity within 5% and controlled the density of Ag NWs by controlling the injection flow of Ag NW ink. The tunable electrical and optical properties of the Ag NW network films were affected by the pumping frequency used to inject Ag NW ink into the slot die. Large-area Ag NW network films with low sheet resistance and high optical transmittance produced by this process are a viable alternative to high-cost ITO films prepared by high-cost vacuum-based sputtering processes. By using diamond-shape patterned Ag NW network films, we obtained highly transparent Ag NW electrodes with a low sheet resistance of 50 Ohm/square, which is acceptable for fabrication of large-area TSPs. Outer/inner, twisting, and rolling test results demonstrated the superior mechanical flexibility of the Ag NW network film to that of conventional ITO films. We further demonstrated the writing operation of flexible TSPs using diamond-patterned Ag NW network electrodes. Together, our results indicate that RTR slot-die-coated Ag NW networks are promising substitutes for conventional ITO electrodes in large-area and low cost flexible TSPs.

## Methods

### Roll-to-roll slot-die coating of Ag NWs and the over layer

400 mm-wide Ag NW network films were prepared on a PET substrate (TORAY ADVANCED MATERIALS KOREA INC.) at room temperature using a specially designed pilot-scale RTR slot-die coating system. The RTR slot die coating system(DKT 2015-R1-SHU500) consisted of ink tanks, a slot die coating zone, a substrate heating zone, and a UV treatment zone. Using the RTR slot die coating system, an Ag NW layer was uniformly coated on a PET substrate through the slot die head and then films were moved to the heating zone (120 °C) by means of unwinding and rewinding at a roller speed of 2 m/min. After coating the Ag NWs, an over-coating layer was uniformly coated on the Ag NW layers to protect the Ag NWs and prevent their degradation. The Ag NWs covered by an over-coating layer were then moved to the heating zone (80 °C) and the UV treatment zone filled with nitrogen ambient.

### Characterization of RTR slot-die coated Ag NW network films

The sheet resistance and resistivity of the Ag NW network films were measured by Hall measurements (HL5500PC, Strength 0.32 T, Accent Optical Technology) at room temperature. The sheet resistance also were measured by tip radius 700 μm of 4-point probe (Model : RSP-1000, DASOLENG in Korea). The optical transmittance of the Ag NW network films was measured by UV/visible spectrometry (Lambda 35) carried out over the wavelength range from 220 to 800 nm. The structural properties of Ag NW network films were analyzed by means of X–ray diffraction (D/MAX-2500). The microstructures and contact region of the Ag NW network electrodes were examined by high-resolution electron microscopy (HRTEM:S-4800Hitachi). Cross-sectional HREM specimen was prepared by means of focus ion beam (FIB) milling. The mechanical properties of OC-Ag NW network films were evaluated using a specially designed inner/outer bending, twisting, and rolling system. In addition, dynamic fatigue bending tests were carried out using a lab-designed cyclic bending test machine, operated at the frequency of 0.5 Hz for 10,000 cycles. The resistances of the Ag NW network films were measured throughout cyclic bending. Furthermore, twisting and rolling tests were carried out using lab-designed twisting and rolling test system at a constant twisting angle of 10° and rolling radius of 70 mm.

### Patterning of Ag NW network films and fabrication of flexible TSPs

Prior to the diamond patterning of OC-Ag NWs films, the OC-Ag NWs films were annealed in oven box at 130 °C for 20 min to prevent film shrinkage. Then, a liquid photo resist (LPR : AZ HKT-601) layer was coated onto the OC-Ag NWs films by a spin-coater. Then, the LPR-coated OC-Ag NW films were exposed to UV light at 60 mJ using a positive diamond mask. The UV-exposed OC-Ag NW films were patterned using developing solution (EN-DT238E: tetramethylammonium hydroxide 3%, surfactant 2%, deionized water 95%). The diamond-patterned OC-Ag NW films were subsequently etched using an etching solution (EO- NS100: nitric acid & deionized water). Finally, the stripped OC-Ag NWs films were cleaned by a spray-type rinse system using deionized water. To fabricate flexible TSPs, Ag paste was directly printed on the diamond-patterned OC-Ag NW film by silk screen printing system. The resulting OC-Ag NW film/OCA/OC-Ag NW film was connected to a flexible printed circuit board by bonding both the metal pattern and the FPCB to an anisotropic conductive film. Both top OC-Ag NW/PET and bottom OC-Ag NW/PET films were bonded to a flexible printed circuit board (FBCB) using an anisotropic conductive film, and then cover glass was attached to the top OC-Ag NW/PET film using OCA film to protect the diamond-patterned top OC-Ag NWs/PET film. Finally, the FPCB was connected to an IC controller to operate the TSPs. TSP fabrication process was explained in supporting information Figure S4.

## Additional Information

**How to cite this article**: Kim, D.-J. *et al.* Roll-to-roll slot-die coating of 400 mm wide, flexible, transparent Ag nanowire films for flexible touch screen panels. *Sci. Rep.* **6**, 34322; doi: 10.1038/srep34322 (2016).

## Supplementary Material

Supplementary Information

## Figures and Tables

**Table 1:** Electrical and optical properties of slot-die coated OC-Ag NW films as function of pumping frequency to control the density of Ag NW network.

Pump frequency	Sheet resistance [Ohm/square]	Transmittance at 550 nm	Average transmittance (380–780)	Haze^1^ [%]	Haze^2^ [%]	Reflection at 550 nm [%]
18 Hz (rpm 525)	30	89%	88%	1.23%	1.04%	10%
11 Hz (rpm 320)	50	90%	89%	0.81%	0.74%	10%
7.5 Hz (rpm 218)	70	90%	90%	0.64%	0.55%	10%

Haze^1^: Ag NW/PET, Haze^2^: Over coating layer/Ag NW/PET.
