# Supplementary material for: Roll-to-roll slot-die coating of 400 mm wide, flexible, transparent Ag nanowire films for flexible touch screen panels
Source: Sci Rep. 2016 Sep 28;6:34322. doi: 10.1038/srep34322 (PMC5039627; doi:10.1038/srep34322)
Supplement: Supplementary Information [file srep34322-s1.pdf]

Additional information

Roll-to-roll slot-die coating of 400 mm wide, flexible, transparent Ag nanowire films for flexible touch screen panels

Dong-Ju Kim[1,2], Hae-In Shin, Eun-Hye Ko, Ki-Hyun Kim, Tae-Woong Kim, and Han-Ki Kim[1,*]

[1]Kyung Hee University, Department of Advanced Materials Engineering for Information and Electronics, 1 Seocheon, Yongin, Gyeonggi-do 446-701, Republic of Korea

[2]Dynamic Korea Technology, R&D Center, 116-60, Sanho-daero, Gumi City, Gyeong-Buk, 39377, Republic of Korea

[3]Samsung Display, OLED R&D Center, Yongin, Gyeonggi-do 446-711, Republic of Korea

[*]imdlhkkim@khu.ac.kr

Roll-to-roll production of an over Coating layer on Ag NW films: Figure S1a shows our pilot–scale RTR slot die coating system (DKT 2015-R1-SHU500) used to coat Ag NWs and over-coating layer onto 125 μm-thick PET substrate (TORAY ADVANCED MATERIALS KOREA INC.). **Table S1** summarized the basic properties of PET substrate used in RTR slot-die coating of the Ag NWs. The RTR slot die coating system consisted of Ag NW ink tanks with a pump, a slot-die coating zone, a substrate heating zone, and a UV treatment zone. To fabricate Ag NWs and the over-coating layer, a slot die coating head (Nordson Corporation, USA) with a width of 600 mm was employed as shown in **Figure S1** a and b. By using an unwinding and rewinding system, the flexible PET substrate was continuously passed through the slot die coating head. In addition, the tension of the flexible PET substrate was controlled by a load cell in the rolling system. PET substrate with a width of 500 mm and thickness of 125 μm was passed over the heating chamber and UV treatment zone as shown in **Figure S1** c and d. The rolling speed of the PET substrate could be exactly controlled by the motor speeds of the unwind and rewind roller. A TACMINA pump with the property of non-pulsation was installed into the RTR coating system as shown in **Figure S1**e. The Ag

NW network density was controlled by pump frequency (Motor RPM).

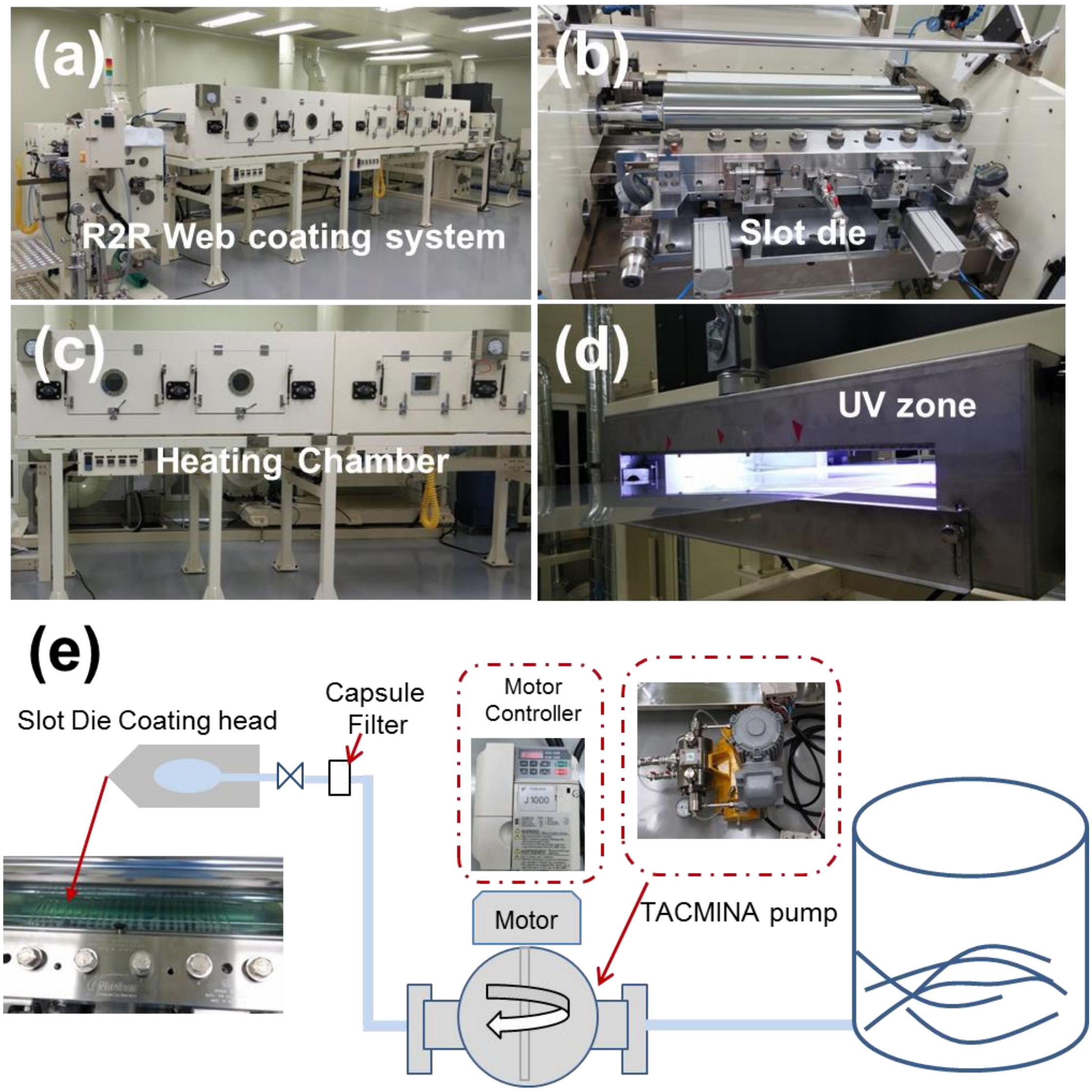

Figure S1. (a) Pictures of the R2R slot-die coating system equipped with an unwinder, rewinder, heating chamber, UV zone, slot die coating head, Ag ink and over-coating ink tank with pump. Picture of (b) slot-die head for Ag NW ink coating, (c) heating chamber to remove solvent in the slot-die coated films, and (d) UV irradiation chamber. (e) Ink supply system for injecting Ag NW ink into the slot die using TACMINA pump.

Figure S1e schematically illustrates process used to coat the Ag NW layer using slot-die coating head with a TACAMINA pump. With increasing the motor rpm, the amount of Ag NW ink coated on the PET substrate was increased through the slot die coating head. The Ag NW layer was coated onto the PET substrate by using a slot die coating head, and then passed

through the heating chamber at 120 ºC by means of unwinding and rewinding at a roller constant speed of 2 m/min (**Figure S1**c). After coating the Ag NW layer, an over-coating layer was coated on the Ag NW layer using the slot-die coating head and passed through the heating chamber at 80 ºC, after which the film was exposed to a UV–mercury type lamp with an intensity of 1000 mJ under a nitrogen ambient by means of unwinding and rewinding rollers at a constant speed of 2 m/min.

Slot-die coating process: In the slot-die apparatus, liquid solution is pumped to the inner part of the slot-die head and ejected through a narrow slot, which is a gap between upstream lip and downstream lip[1,2].

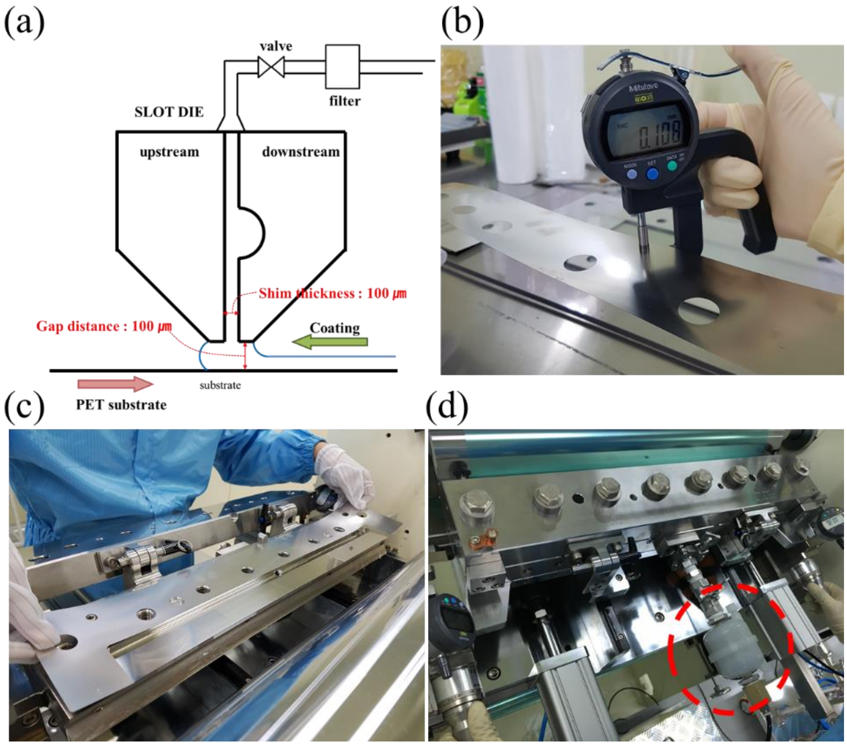

Figure S2. (a) Schematics of slot-die coating head. (b) Picture shows in-situ measured shim thickness (100 μm). Picture of (c) 500 mm-wide shim installed between upstream and downstream lip and (d) capsule filter .

Figure S2a show schematics of the slot-die apparatus for coating of Ag NW ink and over coating layer. In general, the shim, which is injected into the slot-die plays an important role to control the thickness and density of Ag NWs. In our slot-die coating process, we employed a shim with thickness of 100 μm as shown in **Figure 2S**b. The 500 mm wide shim with a thickness of 100 μm was installed between upstream and downstream lips as shown in **Figure S2**c. Finally, we controlled the pressure of Ag NW ink using the capsule filter, which is connected to the inner part of the slot die head, to optimize the uniformity of the Ag NW layer on PET substrate as shown in **Figure S2**d. This capsule filter also remove the bubble in the Ag NW ink.

Diamond-shaped patterning of OC/Ag NWs electrode films: OC-Ag NW films were annealed in box oven at 130 °C for 20 min to prevent film shrinkage as shown in **Figure S3**-a. For diamond-shaped pattering of OC-Ag NWs, a liquid photo resist (LPR : AZ HKT-601) layer was coated onto the OC-Ag NW films by spin coating (NT003_500SA, NST in Korea) as shown in **FigureS3**-b ; operating conditions were a total spin time of 2 min and max spin rpm of 1,500 for 1 min.

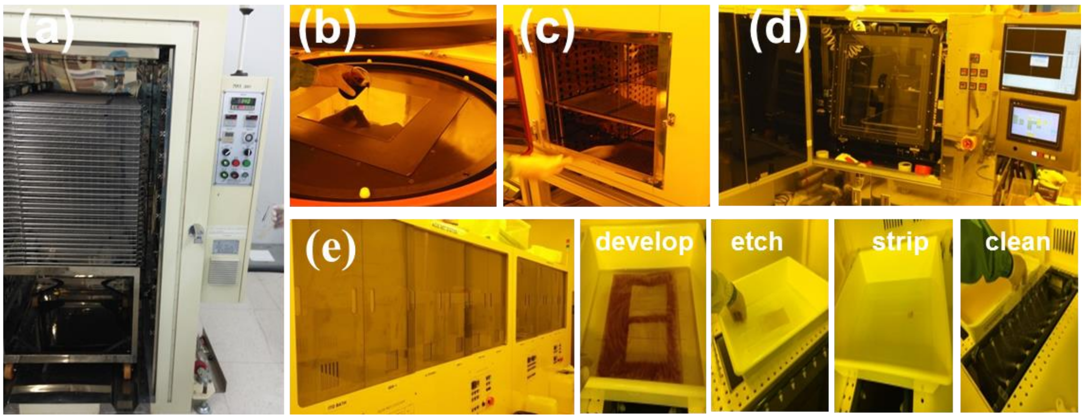

Figure S3. Picture of (a) box oven (b) spin coater, (c) box oven in the yellow room (d) UV exposure, (e) wet station for developer, etcher, striper, and rinsing systems for diamond-shaped patterning of OC- Ag NWs films.

Then, the LPR-coated OC-Ag NW films were annealed at 90°C for 2min to bake in box oven as shown in **Figure S3**-c. The LPR-coated OC-Ag NW films were then exposed to UV light at 60 mJ using a positive diamond mask, as shown in **Figure S3** d. The UV-exposed OC-Ag NW films were patterned by a hand-develop dipping process using a developing solution (EN-DT238E : tetramethylammonium hydroxide 3%, surfactant 2%, deionized water 95%). Diamond-patterned OC-Ag NW films were subsequently etched by a hand-etch dipping process using etching solution (EO- NS100: nitric acid & deionized water). The wet-etched OC-Ag NW films were stripped by a hand-strip dipping process using stripping solution (EN-S800Mo: glycol ethers 10%, sodium gluconate 10%, EDTA 10%, surfactant 5%, deionized water 65%). Finally, the stripped OC-Ag NWs films were cleaned by a spray-type rinse system using deionized water, as shown in **Figure S3**-e.

Fabrication of flexible TSPs : To fabricate flexible TSPs, Ag paste was directly printed on the diamond patterned OC-Ag NW films by silk screen printing System (LDK in Korea) as shown in **Figure S4**-a. The resulting diamond-patterned OC-Ag NW films were used to fabricate film-film-type flexible TSPs and glass-film-film-type TSPs. The top OCO/PET and bottom OCO/PET films were attached to each other using a sheet lamination system(LDK in Korea) and OCA film, as shown in **Figure S4**-b. The resulting OC-Ag NW film/OCA/ OC-Ag NW film were connected to a flexible printed circuit board by bonding both the metal pattern and the FPCB to an anisotropic conductive film as shown in **Figure S4**-c,d. Then, FF-type TSPs were annealed at 50 ºC for 30 min under a pressure of 0.6 MPa to remove the air in the pressure chamber (Auto–Clave: ILSHIN made in Korea) as shown in **Figure S4**-e. **Figure S4-f** shows merged GFF-type TSP. Both top OC-Ag NW/PET and bottom OC-Ag NW/PET films were bonded to a flexible printed circuit board (FBCB) using an anisotropic

conductive film, and then cover glass was attached to the top OC-Ag NW/PET film using OCA film to protect the patterned OC-Ag NW/PET film. Finally, the FPCB was connected to an IC controller to operate the TSPs..

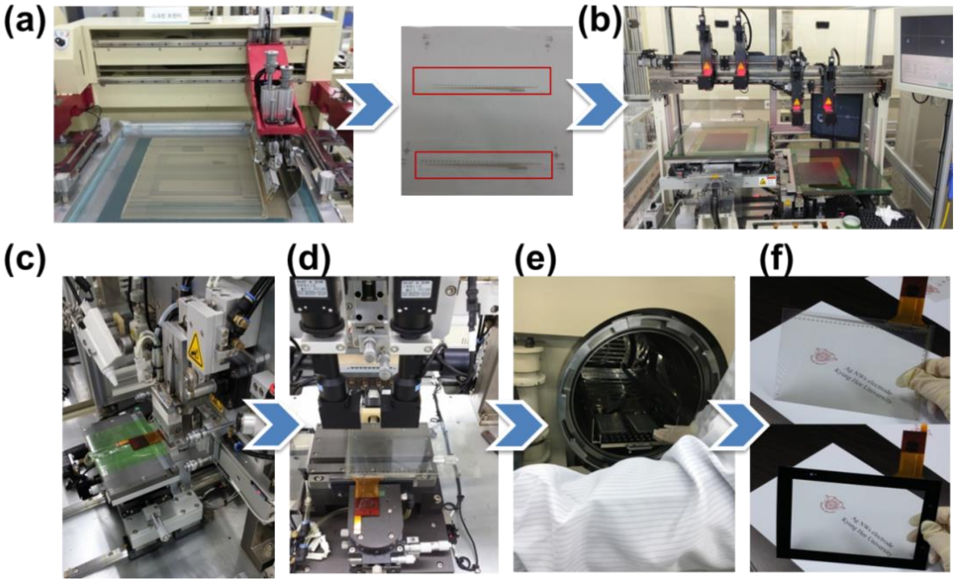

Figure S4. Picture of (a) silk screen printing system, (b) sheet lamination, (c)ACF bonding, (d)FPCB bonding, and (e) the pressure chamber for diamond-shaped patterning of OC Ag NW films and (f)FF & GFF TSPs.

Table S1. Properties of PET film used in roll-to-roll slot-die coating of Ag NWs.

**Item**		**HMD25Z**	**Analysis Method**
Optical Properties	Transmittance	93.3%	JIS K7361(D65) [Nippon Denshoku NDH-2000]
	HAZE	0.17%	
Physical Properties	Adhesion	100/100	JIS D0202-1988 4.15
	Hardness	2H	TAK Method
	Anti-Scratch	Rank 5 (Scratch 0ea)	Steel wool#0000, 2.4kg/12cm^2, 10 times , 20mm/sec
	Water Contact Angle	75°	Water [Kyowa kaimen kagaku, CA-X]

Reference

[1] S.N. Hong, J.J. Lee, H.K. Kang, K.H Lee, Slot-die coating parameters of the low-viscosity bulk-heterojunction materials used for polymer solar cells, Solar Energy Materials & Solar Cells, 112(2013)27.

[2] F.C. Krebs, Fabrication and processing of polymer solar cells: a review of printing and coating techniques, Solar Energy Materials & Solar Cells 93(2009)394.
